# Incidence, Prevalence, and Burden of Health Problems in Elite Female Ice Hockey Players—A One-Season Prospective Study

**DOI:** 10.1155/tsm2/5092272

**Published:** 2025-01-17

**Authors:** Tobias Wörner, Frida Eek

**Affiliations:** ^1^Department of Health Sciences, Lund University, Lund, Sweden; ^2^Department of Molecular Medicine and Surgery, Stockholm Sports Trauma Research Center, Karolinska Institutet, Stockholm, Sweden

**Keywords:** acute injury, epidemiology, illness, overuse, severity

## Abstract

**Introduction:** Epidemiological studies on elite female ice hockey players are lacking but needed to tailor preventive efforts in this growing group of athletes. Therefore, the aim of this study was to describe the incidence, prevalence, and burden of health problems in elite female ice hockey players.

**Methods:** In this prospective cohort study, we asked all Swedish Women's Hockey League (SWHL) players (*N* = 207) to report their health status on the OSTRC-H2 weekly throughout the 2022/2023 season (28 weeks). Reported problems were categorized as injuries (acute or overuse) or illnesses and presented as incidence per player season and mean weekly prevalence.

**Results:** A total of 129 players (62% of all SWHL players) provided 2286 health reports with a mean weekly response rate of 67%. Mean weekly prevalence of health problems was 21% (95% confidence interval [CI]: 19–23) (injuries: 15% [95% CI: 14–17] and illnesses: 6% [95% CI: 5–8]). Injury incidence was 2.1 (95% CI: 1.8–2.4) per player season (acute: 1.2 [95% CI: 1.0–1.5] and overuse: 0.8 [95% CI: 0.7–1.1]). Illness incidence was 1.3 per player season (95% CI: 1.1–1.6). Most reported health problems were acute injuries (59.4% of reported injuries). Most common among acute injuries where to the shoulder (15%), head (13%), and knee (11%). The hip/groin was the most reported (35%) and burdensome (49% of severity score) region among overuse injuries. Reported illnesses were mostly represented by respiratory infections (75%).

**Conclusions:** In average, one in five elite ice hockey players reported a health problem at any given time during the season. Results of this study highlight the need to develop and test primary prevention strategies for shoulder, head, and knee injuries and secondary prevention strategies for hip and groin problems.

## 1. Introduction

Women's ice hockey has been growing exponentially and can be considered as one of the fastest growing sports for female athletes in North America [1–3]. A similar development can be observed in Sweden, where the number of female players has tripled over the past decade (from 3166 in 2010 to > 10,000 in 2024) [4]. In proportion to the rising popularity among women, the scientific attention to this growing group of athletes is underwhelming and studies on the epidemiology of health problems in women's ice hockey are warranted as a first step towards future efforts to preserve players' health.

Most epidemiological data on injuries in female ice hockey come from North American studies that investigate youth [2, 5, 6] or collegiate players [7–9]. The overall incidence of injuries in collegiate female ice hockey players was 5.9 (95% confidence interval [CI] = 5.5–6.3) injuries per 1000 athlete exposures (AEs), with more injuries occurring during competition (11.5/1000 AEs) than in practice (3.7/1000AEs) [10]. Previous studies often define injuries as events leading to time loss from ice hockey (time-loss injury [TL]), which likely captures severe traumatic injuries appropriately [11]. However, athletes may play through injury, which has been reported for traumatic injuries [12] but appears to be especially relevant for overuse injuries [11]. Overuse injuries typically debut gradually and do not necessarily lead to time loss but may still affect performance [13, 14]. Hence, defining an injury only by time loss and expressing its severity by the days lost underestimates the number of injuries occurring in sport.

In recent years, several European studies have been published that comprehensively describe both acute and overuse injuries in male ice hockey players. In these studies, Nordstrøm et al. [15, 16] used the Oslo Sports Trauma Research Center overuse injury questionnaire (OSTRC-H2) [17] to describe all health problems (illnesses, acute, and overuse injuries) in male junior and senior ice hockey players and while also presenting the burden of injuries in ice hockey by putting the injury incidence into the context of their severity as suggested by Bahr, Clarsen, and Ekstrand in 2018 [18]. While acute injuries represent the biggest problem in ice hockey [16], the weekly prevalence of overuse injuries may be close to 50% [19]. To date, no comparable investigation using the OSTRC-H2 to record injuries (acute and overuse) and illnesses has been performed on female ice hockey players.

Even though elite female and male ice hockey players engage in the same sport, there are some differences in game rules that may affect injury patterns. Traditionally, body checking is not permitted in youth and female leagues, while it is part of the game for male players. In Sweden, where our study has been performed, restrictions in body checking have been gradually removed from the women's game in recent years. In 2018, the two top leagues for women in Sweden introduced body checking when fighting for the puck alongside the board. Beginning with the 2022/2023 season, the season our data stems from, body checking in the highest leagues for females is generally permitted all over the rink, except for body checking on open ice while skating in opposite directions (north–south checking) [20]. According to the conceptual model for injury prevention research by van Mechelen et al. [21] the first step in the “sequence of prevention” is to identify the magnitude of the problem. We cannot transfer previous epidemiological results on elite male ice hockey players over to their female counterparts who play hockey under evolving rules. Therefore, a comprehensive investigation of health problems in elite female ice hockey players is urgently needed.

Consequently, the aim of this study was to describe the incidence, prevalence, and burden of health problems (illness and musculoskeletal injuries) in elite female ice hockey players over the course of a season.

## 2. Materials and Methods

In this prospective cohort study, elite female ice hockey players (players in the highest Swedish ice hockey league: Swedish Women's Hockey League [SWHL]) reported the occurrence and burden of health problems throughout the 2022-2023 season (September 2022 to March 2024). The study was approved by the Swedish Ethical Review Authority (2022–02668-01). We followed the *Sports Injury and Illness Surveillance* extension of the Strengthening the reporting of observational studies in epidemiology (STROBE-SIIS) checklist [13] during writing of this manuscript.

### 2.1. Participants and Recruitment

All players listed on the preseason rosters of SWHL teams were eligible for the study. The SWHL is the highest ice hockey league for women in Sweden, including 10 teams geographically spread throughout the country. During preseason (August 2022), we received contact information for all registered players from the SWHL management (*n* = 207). All players were contacted via email and received written information about the study before providing written consent to their participation.

### 2.2. Data Collection

During preseason, participants responded to a baseline survey (digital survey created via survey and report (Artisan Global Media), collecting basic demographic (age and anthropometric data) and ice hockey specific data such as position and experience within ice hockey in general and on current level. Aside from the descriptive data presented in the current study, the baseline survey also included past season injury history, general conditions for playing ice hockey, and assessments of perceived stress, which have been published previously [22, 23]. From September 2022 to April 2023, the participating players received weekly sms invitations via the athlete monitoring system (FitStats Technologies Inc., Moncton, Canada) to report their health status. In the sms invitation, players were asked to report their health status irrespective of absence or presence of health problems. Survey invitations were sent out individually to each player and up to three sms reminders were sent to players not responding. All surveys were sent out as respondent surveys (not anonymized) and data were handled confidentially. Players only received surveys during the ongoing season (regular season + playoffs), which is concluded at different time points due to the playing mode in the SWHL. Hence, once a team finished the season (e.g., not reaching the playoffs or when being eliminated from the playoffs), these players were excluded from new survey postings.

### 2.3. Surveillance Instrument and Definition of Injuries and Illnesses

**Figure 1:**
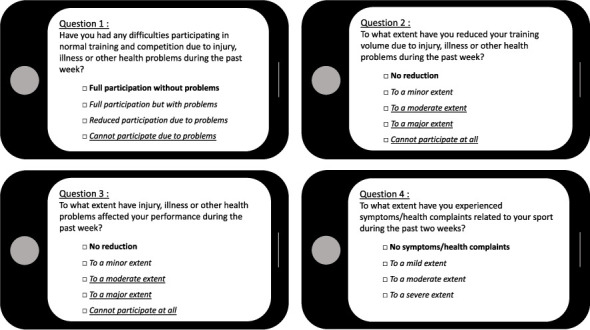
Oslo research center questionnaire on health problems. Players reporting bold responses for all questions were considered not to have health problems. Players reporting at least one of the italic responses in at least one of the four questions were considered to have a health problem. Players reporting any of the italic and underlined responses in any of the questions were considered to have a substantial health problem.

Participating players reported health problems on the Oslo Sports Trauma Research Center Questionnaire on Health Problems (OSTRC-H2) [17, 24]. OSTRC-H2 is a valid surveillance tool and consists of four ordinal questions assessing the (1) ability to participate in training and match play, (2) potential reduction in training volume, (3) effect on performance, and (4) experience of pain—due to an injury or illness during the past week. The minimal answering option to these four questions represent (1) full participation, (2) full training volume, (3) no negative effect on performance, and (4) no pain, thereby indicating for the absence of health problems. We defined health problems (injuries and illnesses) according to the 2020 International Olympic Committee Consensus Statement [13] as “any condition that reduces an athlete's normal state of full health, irrespective of its consequences on the athlete's sports participation or performance or whether the athlete sought medical attention.” Hence, everything but the minimal answering option indicate the presence of a health problem. “Any health problem” was defined by at least one of the four OSTRC-H2 questions answered with anything but the minimal possible answering option. “Substantial health problem” was defined as problems with at least moderate to severe reductions in training volume and/or performance and/or a complete inability to participate in ice hockey practice or games [17] (Figure 1).

Injuries were categorized as acute or overuse injuries. “Acute injuries” were defined by a sudden symptom debut during a single, identifiable injury event and “overuse injuries” by a gradual onset of symptoms in absence of such a single, identifiable event. Players also reported the anatomical location for the injury and perceived occurrence/mechanisms for acute injuries. “Illnesses” were defined as health problems not related to the musculoskeletal system and categorized according to affected organ system or symptoms [13]. If several different symptoms were reported by the player related to the same illness report, we categorized it as “multiple organ systems. If general symptoms that could not be fitted to an organ system were reported, we classified those as ”not specified.” For every health problem (injuries and illnesses), players were asked to report the number of days they missed practice or games (time loss). Multiple health problems could also be reported before the weekly survey was concluded. Players also reported their exposure to games (count) and practice (hours) during the past week.

### 2.4. Data Management and Statistical Analysis

Raw data were extracted from the athlete monitoring system and handled in Microsoft Excel and SPSS Statistics 27 (IBM). Severity scores were based on OSTRC (range 0 [full participation without health problems] to 100 [could not participate due to a health problem]) [24] and calculated for each weekly reported health problem. An individual health problem was defined as a health problem that either was reported only in a single week or reported continuously over several consecutive weeks. Associated time loss and severity scores for each individual health problem were summed up for all weeks the health problem was ongoing/reported.

Time under risk was primarily expressed by player season, calculated as the sum of all weekly responses (weeks with missing responses not included) divided by the total length of the season (28 weeks/196 days). Incidence of health problems was calculated by dividing the total number of individual health problems by the number of players season and presented with 95% CIs (the Poisson 95% CI for the number counted [the numerator]), calculated via MedCalc Software Ltd (CI for a rate: https://www.medcalc.org/calc/rate_ci.php [Version 23.0.2])

The incidence of acute injuries was further calculated based on reported occurrence during practice (all/no specific forms of training) vs. games (all/no specific forms of games) with time under risk summarized as total practice hours vs. total game count (incidence rate presented as acute injury/1000 practice hours vs. acute injuries/1000 game count). The prevalence of different health problems during the season was calculated and presented for each week and as average weekly prevalence for the whole season (sum of weekly prevalence divided by 28 weeks, alongside the belonging 95% CI). Burden of a health problem is presented as the cross product of the incidence and severity of individual health problems.

As a complement, we also presented a comparison of incidence between subgroups based on total summarized response rates (1–9 weeks [< 35%]; 10–20 weeks [35% to < 75%]; and 21+ weeks [75%–100%]) over the course of the season to investigate potential effects of nonresponse on our results.

## 3. Results

Among the 207 invited SDHL players, we received 170 baseline responses (82%). Of these 170 players, 129 (76%, respective 62% of all active players in the league) answered at least once to the weekly health surveillance and were included in the final study sample. Characteristics of the final sample are described in Table 1.

### 3.1. Weekly Response Rates to OSTRC-H2

**Figure 2:**
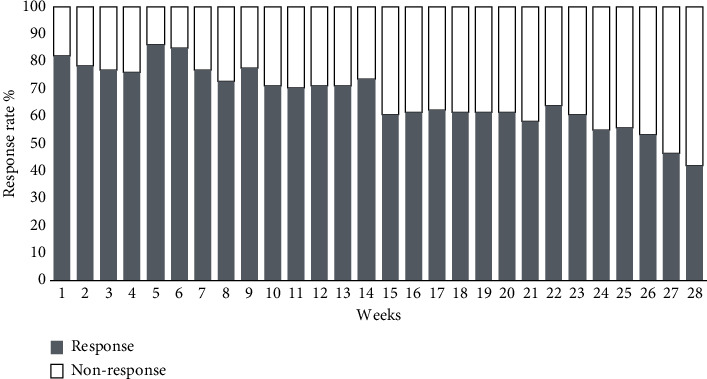
Response rates to weekly surveillance throughout the season.

We send out 3367 surveys and received 2286 responses (68%) over the course of 28 weeks (mean weekly response rate: 66.8% and range: 42%–86%). The mean response rate was higher during the regular season (week: 1–23) than in the play offs (week: 24–28) (67.2%; range: 58%–86% vs. 50.4%; range: 42%–56%) (Figure 2). The total summarized primary time under risk (player seasons) was 81.64 player seasons. In total, 22,763 training hours and 3492 games were reported.

### 3.2. Number, Incidence, and Severity of Health Problems

Over the course of the season, in total 277 health problems were reported. The players reported 170 injuries (59.4% [*n*: 101] acute injuries and 40.6% [*n* = 69] overuse injuries), and 107 illnesses. The injury incidence was 2.1 (95% CI: 1.8–2.4) per player season (acute injuries: 1.2 [95% CI: 1.0–1.5] per player season and overuse injuries: 0.8 [95% CI: 0.7–1.1] per player season). Acute injuries occurred more frequently during games (incidence per 1000 games [95% CI]: 21.5 [16.9–26.9]) than during practice (incidence per 1000 practice hours [95% CI]: 0.72 [0.43–1.13]). Incidence of illnesses was 1.3 (95% CI: 1.1–1.6) per player season. The subgroup comparison of incidence rates between players with different response rates showed relatively higher rates among players with lower response rates. There was, however, no obvious effect on the total incidence rates, which remained at a relatively similar level to the group with a high response rate (Table 2). The total time loss due to health problems was on average 11.4 days per player season. Time loss from injuries was 7.2 days per player season (acute injuries: 6.1 days per player season and overuse injuries: 1.1 days per player season). Time loss from illnesses was 4.3 days per player season.

Among acute injuries (*n* = 101), 75 occurred during games and 19 during practice (7 acute injuries during other occasions). Eighty-one injuries were caused by collisions, either with an object (*n* = 39) or another player (*n* = 42). The remaining injuries resulted from jump/landing (*n* = 4), change of direction/cutting (*n* = 2), sprint/rush (*n* = 1) skill-related actions without contact (*n* = 1), or other causes (*n* = 12). Among the 75 injuries that occurred during games, 36 (48%) were due to collisions with another player, and 33 (44%) were due to collisions with an object. The remaining injuries occurred due to change of direction/cutting (*n* = 2; 3%) or other causes (*n* = 4; 5%). Of the 19 injuries that occurred during practice, 6 (32%) were caused by collisions with another player and 6 (32%) by collisions with an object. The remaining injuries were due to sprinting/rushing (*n* = 1), skill-related actions without contact (*n* = 1), or other causes (*n* = 5).

**Figure 3:**
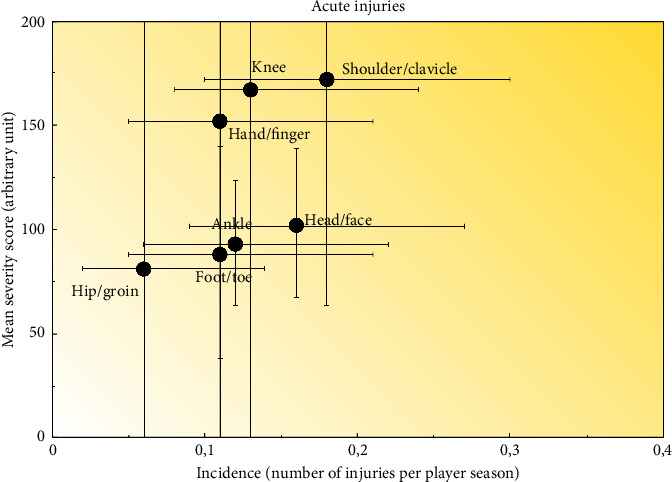
Risk matrix illustrating the relationship between incidence and severity of reported acute injuries. Horizontal lines represent 95% CI for incidence. Vertical lines represent 95% CI for severity scores. Only injuries that were reported at least 5 times during the season are included. The gradient indicates injury burden (the darker the color, the greater the burden of injury).

**Figure 4:**
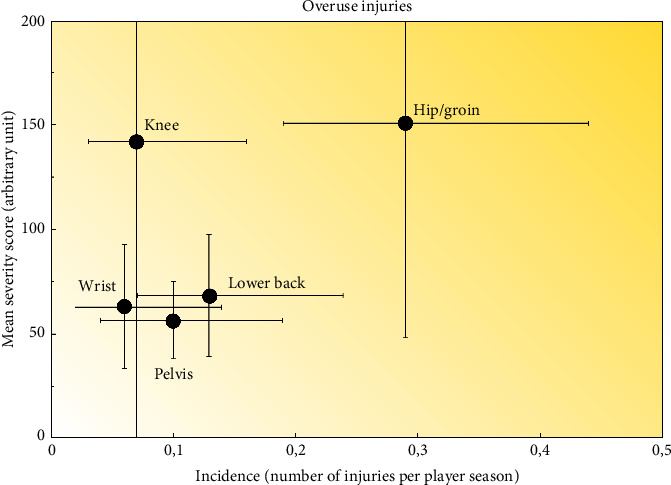
Risk matrix illustrating the relationship between incidence and severity of reported overuse injuries. Horizontal lines represent 95% CI for incidence. Vertical lines represent 95% CI for severity scores. Only injuries that were reported at least 5 times during the season are included. The gradient indicates injury burden (the darker the color, the greater the burden of injury).

The shoulder, head/face, and knee regions (*n* = 15 [14.8%], *n* = 13 [12.8%], and *n* = 11 [10.8%]) were the most frequently reported. Among overuse injuries (*n* = 69), the hip/groin, lower back, and pelvis regions (*n* = 24 [34.7%], *n* = 11 [15.9%], and *n* = 8 [11.6%]) were most frequently reported. Hip and groin injuries represented 49% of the total severity score among overuse injuries. The respiratory system was most frequently affected among reported illnesses (71%). Twenty-one percent of reported illnesses were a combination of different organ systems, also dominated by respiratory problems as primary illness, followed by few counts of other systems such as gastrointestinal and mental illnesses (Table 3). Injury incidence in relation to injury severity is illustrated in Figure 3 (acute injuries) and Figure 4 (overuse injuries).

### 3.3. Prevalence of Health Problems

**Figure 5:**
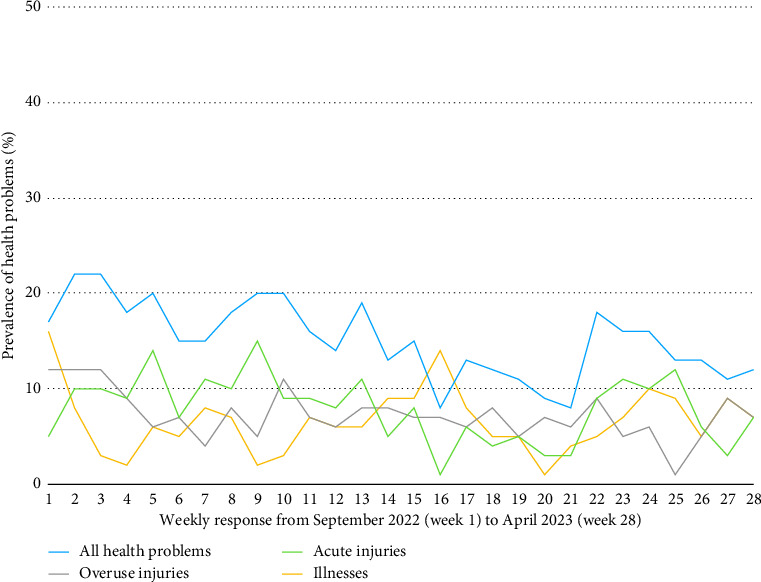
Weekly prevalence of health problems over the course of the season.

**Figure 6:**
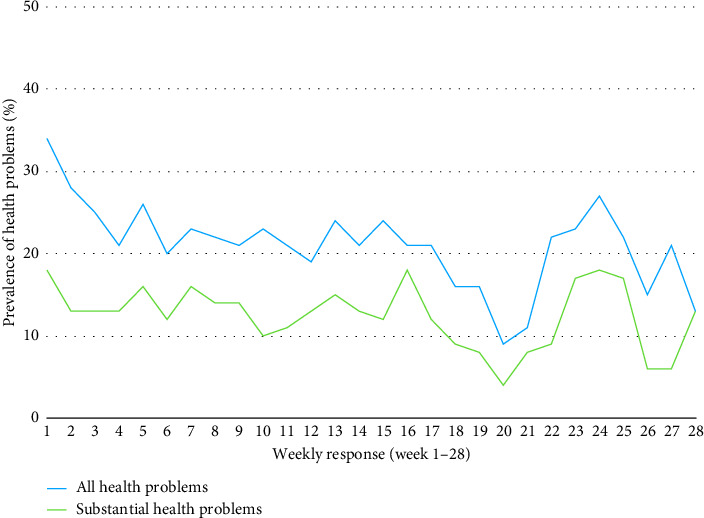
Weekly prevalence of all and substantial health problems over the course of the season.

The average weekly prevalence of health problems was 21% (95% CI: 19–23). Fifteen percent of weekly health problems were injuries (95% CI: 14–17) and 7% (95% CI: 5–8) illnesses. The average weekly prevalence for substantial and nonsubstantial health problems, categorized into injuries (acute and overuse) and illnesses is summarized in Table 4 and illustrated in Figure 5. The weekly prevalence of injuries (acute and overuse) and illnesses is illustrated in Figure 6.

## 4. Discussion

This study investigated incidence, prevalence, and burden of health problems among elite female ice hockey players. In our study, acute injuries had the highest incidence (2.1/player season), followed by illnesses (1.3/player season), and overuse injuries (0.8/player season). The mean weekly prevalence of health problems was 21%, two-thirds of this prevalence was due to injuries. The mean weekly prevalence of substantial health problems was 12%. Shoulder and knee injuries were the most burdensome among acute injuries while injuries in the hip and groin region represented 49% of the total severity score among overuse injuries.

Our study is the first to use OSTRC-H2 [24] to report health problems in elite female ice hockey players while the existing epidemiological literature focuses on youth and collegiate players [2, 7, 8, 10, 25]. Therefore, there are no other studies with the same target population to compare our results with. However, we can compare our findings to recent studies using similar methodology on male ice hockey players [15, 16, 19]. The reported incidence of injuries and illnesses in female elite ice hockey players appears to be substantially lower than in male junior and senior elite ice hockey players [15, 16]. Since Nordstrøm et al. [15, 16] expressed their injury rate per athlete year (per athlete-year), while we express our rate per player season, the difference between our observations may even be an underestimation. Hence, elite female ice hockey players appear to be at lower risk for injury than their male counterparts with OSTRC-H2 used as reporting method, similar to other research using different reporting methods and injury definitions [3].

In accordance with previous studies on male elite ice hockey players with similar surveillance method [15, 16], injuries to the shoulder, knee, head/face, and ankle were most the frequently reported acute injuries. The nature of ice hockey being fast paced and associated with physical contact makes shoulder and head injuries quite expectable. However, in contrast to our findings, previous research found female players to be more prone to lower extremity injuries than upper extremity injuries [3, 26]. This difference in injury patterns is often attributed to differences in contact rules for women and men. However, here in Sweden, body checking rules have been aligned among top leagues for women and men over the past years as specified in the introduction. Nevertheless, a cross-sectional study on elite female and male ice hockey players in Sweden, based on injuries during the 2021/2022 season, found comparable injury patterns between sexes [22]. Consequently, not just the rules of the game but also the pattern of injury occurrence appears to be similar between Swedish elite ice hockey players and elite male players. Trauma to the head, especially concussions, which are common in ice hockey [27], are a particular concern when reflecting on the change of bodychecking rules in Swedish elite female hockey. Sports-related concussion, defined as “*a traumatic brain injury caused by a direct blow to the head, neck, or body*” [28], is straightforwardly related to body contact, which is why a recent consensus statement supports rule changes that disallow body checking [28]. In child and adolescent hockey leagues, disallowing of body checking is associated with substantial lower practice-related concussion rates (IRR 0.42) compared with leagues that allow bodychecking [29]. Despite differences in bodychecking rules, other studies have found higher rates of concussions in female players compared with male players [1, 3, 30] and the main injury mechanism in hockey is contact with other players [3]. With its changes in bodychecking rules, the SWHL aspires to improve performance and competitiveness, while reducing the number of concussions [20]. A recent cross-sectional study, comparing preseason injury prevalence among Swedish elite female and male players, found comparable proportions of head injuries among reported injuries [22]. Yet, our data indicate that head/face injuries are among the most common injuries in female players, ranking third in terms of time loss and severity. Unfortunately, we do not have previous data from Sweden to compare our findings with. Whether or not rule changes will affect the rate of concussions in Swedish female elite hockey should be investigated prospectively [28].

Hip and groin injuries were the most frequently reported overuse injuries and accounted for 49% of the total severity score of injuries with gradual onset. Groin injuries are reported to be more common in elite male than elite female athletes [31], but a recent study on Swedish elite ice hockey players reported comparable seasonal prevalence of hip and groin problems between sexes [22]. In the study by Wörner, Kauppinen, and, Eek [22], the OSTRC-O [17] was modified to collect seasonal prevalence and severity (injuries leading to decreased training volume/ability to participate in ice hockey as well as performance impairments), and hip/groin injuries were found to be more severe among female players. Our current prospective data strengthen these findings by showing that injuries in the hip and groin regions are the most burdensome overuse injuries in elite women's ice hockey. The general overuse injury pattern with the hip and groin, lumbo-pelvic region, as major players is consistent with previous studies on elite male players [15, 16, 19]. While primary prevention strategies for groin problems in form of eccentric strengthening protocols have been proven effective in other sports such as football [32], they have not been tested in ice hockey. Continuous monitoring of hip and groin health with the goal to identify existing problems early to subsequently act upon them has been suggested as secondary prevention strategy in football players [33]. Similar secondary prevention strategies may be applied and should be tested in ice hockey by using screening tests such as the 5-s squeeze test [34]. A recent study with the small sample size indicates that cam morphology might be highly prevalent among elite female ice hockey players [35] but the prevalence of femoroacetabular impingement or other causes of hip-related pain in elite ice hockey players has not yet been investigated.

Accounting for 71% of all reported illnesses, the respiratory system was most affected in our study, which is consistent with studies on junior and senior male ice hockey players [15, 16]. Interestingly, the studies by Nordstrøm et al. [15, 16] predated the COVID-19 pandemic. The COVID-19 pandemic has taught us the importance of preventive measures to reduce the risk for an infection. During the winter games in PyeongChang in 2018, 194 illnesses (71% of all illnesses) affecting the respiratory system were recorded [36]. In the 2022 Beijing games, an event held during a national lockdown and with extensive preventive measures in place, the total number of respiratory illnesses was 52 (48%) [37]. Hence, it is somewhat surprising that our data are comparable to prepandemic data on similar populations [15, 16]. However, other external factors may play an important role in explaining the high numbers of reported respiratory symptoms. Professional ice hockey is played indoors and with varying air quality. Players inspire cold and dry air at high which leads to cooling and dehydration of the airway's surface area [38], which is why respiratory symptoms are prevalent in athletic populations such as cross-country skiers [39]. According to our data, illnesses represent the second largest burden on female ice hockey players (second to acute injuries) and their prevention should hence be one of the future objectives of governing bodies, clubs, and players. In a recent study, describing mental health in elite female ice hockey players, it was reported that 6 out of 10 players in the SWHL do not reach the ideal state of mental health and that mental health problems such as anxiety, sleeping problems, and depression were highly prevalent [40]. Our prospective data do not confirm these findings, even though our data collection tool includes symptoms of mental health. Timing of the cross-sectional data collection by Johansson et al. [40], just before the play offs, as well as the use of multiple self-reporting outcomes specifically developed for mental illnesses, may have contributed to the high prevalence recorded in their study. OSTRC-H2 includes mental illness as a symptom and may have underestimated the incidence and prevalence of mental illness in our study. Therefore, future prospective studies should further investigate mental illness in elite ice hockey players.

Most existing studies in ice hockey have primarily focused on the first two steps of the four steps in Van Mechelen's “sequence of prevention” model [21], namely, the extent, etiology, and mechanisms of injuries. A systematic review compiled studies evaluating interventions aimed at reducing aggression-related injuries in ice hockey, finding that changes to mandatory rules were associated with reductions in such injuries [41]. However, there are currently few studies that have explored the application of preventive physical interventions targeting specific body regions, with the exception of a 2002 study investigating the prevention of adductor strains [42]. This highlights an important area for further development in ice hockey research.

### 4.1. Methodological Considerations

This is the first study investigating health problems in female elite ice hockey players, using recommended data collection, and reporting methods [13]. With 82% of the total study population responding to the initial invitation to participate, generalizability to elite female ice hockey players was promising. Unfortunately, the final sample included 62% of the total population and potential nonresponse bias cannot be excluded. Furthermore, lower continuous response rates among participants during the season may lead to an overestimation of incidence rates [43]. However, we do not believe that nonresponses can be assumed to represent the absence of health problems. Therefore, we decided to calculate the time under risk only based on weeks during which players responded. As presented in Table 2, a subgroup comparison of incidence rates between players with different response rates showed in general higher an incidence among low responders. However, we did not see an obvious effect on results from our total sample. Furthermore, presented results such as weekly prevalence and distribution of health problems is reported to be less affected by low response rates [43]. All health problems were reported by the players and the OSTRC-H2 only provides injury categorized symptoms according to anatomical location and organ system, so our study lacks specific clinical diagnoses. Self-reporting has both advantages and disadvantages. One advantage is that it allows athletes to report a wide range of injuries and issues, even in the absence of formal clinical evaluation or records. However, self-reports do not provide detailed information about formal diagnoses or medical assessments. In addition, athletes may find it challenging to distinguish between acute and overuse injuries, for example, when pain appears suddenly, even though underlying tissue changes may have been developing over time due to overuse or inadequate recovery. Future studies are needed to shine light on e.g., underlying causes of groin pain [44, 45] or to separate concussions from other reported head/face problems in elite ice hockey players.

## 5. Conclusion

This study showed that on average, one in every five elite female ice hockey players reported a health problem at any given time during the season. Acute injuries were the most common health problem with the shoulder, head, and knee regions being most frequently affected. Primary prevention of injuries to these anatomical areas should hence be in the center of future investigations and on-field efforts. Overuse injuries in the hip and groin regions stood for half of the total burden among all overuse injuries and had the highest severity scores among all injuries. Hence, secondary prevention strategies for hip and groin problems are recommended to be prioritized among female ice hockey players.

## Figures and Tables

**Table 1:** Player characteristics (*n* = 129).

Demographics (mean [SD])	
Age in years	23.1 (4.5)
Weight in kg	68.7 (6.8)
Height in cm	168.5 (11.6)
Ice hockey experience in years mean (SD)	
In total	16.8 (4.5)
On current level	5.2 (4.5)
Playing position % (*n*)	
Goaltender	12.5 (16)
Defense	36.7 (47)
Forward	51.2 (66)
Practice hours/week (mean [SD])	10.1 (4.9)
Median (IQR) min–max	10 (7–12) 0–30
Games/week (mean [SD])	1.6 (1.0)
Median (IQR) min–max	2 (1–2) 0–7

Abbreviations: cm = centimeter, IQR = inter quartile range (quartile1–quartile3), kg = kilogram, SD = standard deviation.

**Table 2:** Subgroup comparison of incidence rates among participants with low, medium, and high weekly response rates.

	Total sample (*n* = 129)	High response rate (*n* = 67)	Medium response rate (*n* = 29)	Low response rate (*n* = 33)
Total time under risk (player seasons)	81.6	61.2	14.6	5.9
Injury incidence/player season (95% CI)	2.1 (1.8–2.4)	1.9 (1.6–2.3)	2.3 (1.5–3.1)	3 (1.8–4.7)
Illness incidence/player season (95% CI)	1.3 (1.1–1.6)	1.3 (1.0–1.6)	0.9 (0.5–1.5)	2.5 (1.4–4.1)
Acute injuries				
/1000 practice hours (95% CI)	0.72 (0.43–1.13)	0.95 (0.54–1.55)	0.24 (0.01–1.3)	1.17 (0.1–4.2)
/1000 games (95% CI)	21.5 (16.9–26.9)	20.2 (15.2–26.4)	23.5 (12.8–39.4)	30.1 (12.5–63.8)

*Note:* High response rate = responded for 21+ weeks (75%–100% of the study period). Medium response rate = responded for 10–20 weeks (35%–75% of the study period). Low response rate = responded for 1–9 weeks (4%–35% of the study period).

**Table 3:** Amount of time loss and total severity scores for all health problems.

Health problem (*n*)	Total days of time loss	Number of injuries according to categorized time loss (days)				Severity score
	Days	Slight (0)	Mild (0–7)	Moderate (8–28)	Severe (> 28)	Sum
Injury (170)	585	88	62	17	3	19,663
Acute (101)	498	41	44	13	3	12,199
Shoulder/clavicle (15)	90	6	5	3	1	2591
Head/face (13)	59	1	11	1	0	1336
Knee (11)	94	6	3	1	1	1838
Ankle (10)	49	1	8	1	0	936
Foot/toe (9)	30	5	2	2	0	797
Hand/finger (9)	48	6	1	2	0	1372
Hip/groin (5)	19	2	2	1	0	408
Neck (4)	6	1	3	0	0	230
Forearm (4)	34	2	1	0	1	665
Abdomen (4)	5	1	3	0	0	276
Thigh (4)	2	3	1	0	0	180
Elbow (3)	1	2	1	0	0	166
Pelvis (3)	6	2	1	0	0	191
Lower back (2)	6	1	1	0	0	173
Lower leg (2)	21	1	0	1	0	469
Wrist (1)	27	0	0	1	0	420
Chest (1)	0	1	0	0	0	92

Overuse (69)	87	48	18	3	0	7464
Hip/groin (24)	49	15	6	3	0	3645
Lower back (11)	6	8	3	0	0	752
Pelvis (8)	7	4	4	0	0	450
Knee (6)	5	4	2	0	0	853
Wrist (5)	1	4	1	0	0	316
Neck (3)	0	3	0	0	0	175
Shoulder (3)	11	2	1	0	0	256
Abdomen (3)	0	3	0	0	0	114
Forearm (2)	0	2	0	0	0	40
Thigh (2)	7	1	1	0	0	141
Hand/fingers (1)	1	1	0	0	0	25
Chest (1)	0	1	0	0	0	697

Illness (107)	348	24	75	7	1	9528
Respiratory (71)	229	13	53	5	0	5979
Gastrointestinal (3)	5	0	3	0	0	177
Psychological (2)	4	1	1	0	0	274
Multiple systems (20)	79	6	12	1	1	2154
Unknown (11)	31	4	6	1	0	974

**Table 4:** Mean weekly prevalence of all and substantial health problems over the course of the season (28 weeks).

Weekly prevalence	Mean (%)	95% CI	Range
All health problems	21	19–23	9–34
Injuries	15	14–17	8–22
Acute injuries	8	7–9	1–15
Overuse injuries	7	6–8	1–12
Illnesses	7	5–8	1–16
Substantial health problems	12	11–14	4–18
Injuries	8	7–9	3–14
Acute injuries	5	4–6	0–11
Overuse injuries	3	2–4	0–6
Illnesses	5	4–6	0–11

Abbreviation: 95% CI = 95% confidence interval.

## Data Availability

The data that support the findings of this study are available on request from the corresponding author. The data are not publicly available due to privacy or ethical restrictions.

## References

[1] Abbott K. (2014). Injuries in Women’s Ice Hockey: Special Considerations. *Current Sports Medicine Reports*.

[2] Decloe M. D., Meeuwisse W. H., Hagel B. E., Emery C. A. (2014). Injury Rates, Types, Mechanisms and Risk Factors in Female Youth Ice Hockey. *British Journal of Sports Medicine*.

[3] MacCormick L., Best T. M., Flanigan D. C. (2014). Are There Differences in Ice Hockey Injuries Between Sexes?: A Systematic Review. *Orthopaedic Journal of Sports Medicine*.

[4] Milestone Reached–10,000 Registered Girls and Women Players in Sweden. https://www.swehockey.se/tiotusen-damspelare.

[5] Emery C. A., Meeuwisse W. H. (2006). Injury Rates, Risk Factors, and Mechanisms of Injury in Minor Hockey. *The American Journal of Sports Medicine*.

[6] Roberts W. O., Brust J. D., Leonard B. (1999). Youth Ice Hockey Tournament Injuries: Rates and Patterns Compared to Season Play. *Medicine and Science in Sports and Exercise*.

[7] Agel J., Dick R., Nelson B., Marshall S. W., Dompier T. P. (2007). Descriptive Epidemiology of Collegiate Women’s Ice Hockey Injuries: National Collegiate Athletic Association Injury Surveillance System, 2000-2001 Through 2003-2004. *Journal of Athletic Training*.

[8] Agel J., Harvey E. J. (2010). A 7-Year Review of Men’s and Women’s Ice Hockey Injuries in the NCAA. *Canadian Journal of Surgery*.

[9] Schick D. M., Meeuwisse W. H. (2003). Injury Rates and Profiles in Female Ice Hockey Players. *The American Journal of Sports Medicine*.

[10] Chandran A., Nedimyer A. K., Boltz A. J., Robison H. J., Collins C. L., Morris S. N. (2021). Epidemiology of Injuries in National Collegiate Athletic Association Women’s Ice Hockey: 2014-2015 Through 2018-2019. *Journal of Athletic Training*.

[11] Clarsen B., Bahr R. (2014). Matching the Choice of Injury/Illness Definition to Study Setting, Purpose and Design: One Size Does Not Fit All. *British Journal of Sports Medicine*.

[12] Hammond L. E., Lilley J. M., Pope G. D., Ribbans W. J. (2014). The Impact of Playing in Matches While Injured on Injury Surveillance Findings in Professional Football. *Scandinavian Journal of Medicine and Science in Sports*.

[13] Bahr R., Clarsen B., Derman W. (2020). International Olympic Committee Consensus Statement: Methods for Recording and Reporting of Epidemiological Data on Injury and Illness in Sport 2020 (Including STROBE Extension for Sport Injury and Illness Surveillance (STROBE-SIIS)). *British Journal of Sports Medicine*.

[14] Worner T., Clarsen B., Thorborg K., Eek F. (2019). Elite Ice Hockey Goalkeepers Have a High Prevalence of Hip and Groin Problems Associated With Decreased Sporting Function: A Single-Season Prospective Cohort Study. *Orthopaedic Journal of Sports Medicine*.

[15] Nordstrom A., Bahr R., Clarsen B., Talsnes O. (2021). Prevalence and Burden of Self-Reported Health Problems in Junior Male Elite Ice Hockey Players: A 44-Week Prospective Cohort Study. *The American Journal of Sports Medicine*.

[16] Nordstrom A., Bahr R., Talsnes O., Clarsen B. (2020). Prevalence and Burden of Health Problems in Male Elite Ice Hockey Players: A Prospective Study in the Norwegian Professional League. *Orthopaedic Journal of Sports Medicine*.

[17] Clarsen B., Ronsen O., Myklebust G., Florenes T. W., Bahr R. (2014). The Oslo Sports Trauma Research Center Questionnaire on Health Problems: A New Approach to Prospective Monitoring of Illness and Injury in Elite Athletes. *British Journal of Sports Medicine*.

[18] Bahr R., Clarsen B., Ekstrand J. (2018). Why We Should Focus on the Burden of Injuries and Illnesses, Not Just Their Incidence. *British Journal of Sports Medicine*.

[19] Brunner R., Bizzini M., Niedermann K., Maffiuletti N. A. (2020). Epidemiology of Traumatic and Overuse Injuries in Swiss Professional Male Ice Hockey Players. *Orthopaedic Journal of Sports Medicine*.

[20] Tacklingar Införs I Sdhl Och Damettan (Body Checking Is Introduced in the SDHL and the Women’s Division 1). https://www.sdhl.se/article/633val3bc-5nfcdd/view.

[21] van Mechelen W., Hlobil H., Kemper H. C. (1992). Incidence, Severity, Aetiology and Prevention of Sports Injuries. A Review of Concepts. *Sports Medicine*.

[22] Worner T., Kauppinen S., Eek F. (2023). Injury Patterns in Swedish Elite Female and Male Ice Hockey—A Cross-Sectional Comparison of Past-Season’s Injuries. *Physical Therapy in Sport: Official Journal of the Association of Chartered Physiotherapists in Sports Medicine*.

[23] Worner T., Kauppinen S., Eek F. (2024). Same Game, Different Worlds? General Conditions, Perceived Stress, and Associations Between Stress and Past Season Injuries in Elite Female and Male Ice Hockey Players. *BMC Sports Science, Medicine and Rehabilitation*.

[24] Clarsen B., Bahr R., Myklebust G. (2020). Improved Reporting of Overuse Injuries and Health Problems in Sport: An Update of the Oslo Sport Trauma Research Center Questionnaires. *British Journal of Sports Medicine*.

[25] Dryden D. M., Francescutti L. H., Rowe B. H., Spence J. C., Voaklander D. C. (2000). Epidemiology of Women’s Recreational Ice Hockey Injuries. *Medicine and Science in Sports and Exercise*.

[26] Tuominen M., Stuart M. J., Aubry M., Kannus P., Tokola K., Parkkari J. (2016). Injuries in Women’s International Ice Hockey: an 8-Year Study of the World Championship Tournaments and Olympic Winter Games. *British Journal of Sports Medicine*.

[27] Prien A., Grafe A., Rossler R., Junge A., Verhagen E. (2018). Epidemiology of Head Injuries Focusing on Concussions in Team Contact Sports: A Systematic Review. *Sports Medicine*.

[28] Patricios J. S., Schneider K. J., Dvorak J. (2023). Consensus Statement on Concussion in Sport: The 6th International Conference on Concussion in Sport-Amsterdam, October 2022. *British Journal of Sports Medicine*.

[29] Eliason P. H., Galarneau J. M., Kolstad A. T. (2023). Prevention Strategies and Modifiable Risk Factors for Sport-Related Concussions and Head Impacts: A Systematic Review and Meta-Analysis. *British Journal of Sports Medicine*.

[30] Eckner J. T., O’Connor K. L., Broglio S. P., Ashton-Miller J. A. (2018). Comparison of Head Impact Exposure Between Male and Female High School Ice Hockey Athletes. *The American Journal of Sports Medicine*.

[31] Orchard J. W. (2015). Men at Higher Risk of Groin Injuries in Elite Team Sports: A Systematic Review. *British Journal of Sports Medicine*.

[32] Haroy J., Clarsen B., Wiger E. G. (2019). The Adductor Strengthening Programme Prevents Groin Problems Among Male Football Players: A Cluster-Randomised Controlled Trial. *British Journal of Sports Medicine*.

[33] Wollin M., Thorborg K., Welvaert M., Pizzari T. (2018). In-Season Monitoring of Hip and Groin Strength, Health and Function in Elite Youth Soccer: Implementing an Early Detection and Management Strategy Over Two Consecutive Seasons. *Journal of Science and Medicine in Sport/Sports Medicine Australia*.

[34] Worner T., Thorborg K., Eek F. (2019). Five-Second Squeeze Testing in 333 Professional and Semiprofessional Male Ice Hockey Players: How Are Hip and Groin Symptoms, Strength, and Sporting Function Related?. *Orthopaedic Journal of Sports Medicine*.

[35] Ross K. A., Mojica E. S., Lott A., Carter C., Gonzalez-Lomas G. (2023). Characterization of Pincer-Type Hip Impingement in Professional Women’s Ice Hockey Players. *The Physician and Sportsmedicine*.

[36] Soligard T., Palmer D., Steffen K. (2019). Sports Injury and Illness Incidence in the PyeongChang 2018 Olympic Winter Games: A Prospective Study of 2914 Athletes From 92 Countries. *British Journal of Sports Medicine*.

[37] Soligard T., Palmer D., Steffen K. (2023). Olympic Games During Nationwide Lockdown: Sports Injuries and Illnesses, Including COVID-19, at the Beijing 2022 Winter Olympics. *British Journal of Sports Medicine*.

[38] Anderson S. D., Kippelen P. (2008). Airway Injury as a Mechanism for Exercise-Induced Bronchoconstriction in Elite Athletes. *The Journal of Allergy and Clinical Immunology*.

[39] Bernhardsen G. P., Stang J., Halvorsen T., Stensrud T. (2023). Differences in Lung Function, Bronchial Hyperresponsiveness and Respiratory Health Between Elite Athletes Competing in Different Sports. *European Journal of Sport Science*.

[40] Johansson H., Malmborg J. S., Ekengren J., Lind J., Ivarsson A. (2023). Skating on Thin Ice? Mental Health and Well-Being in Women’s Ice Hockey. *BMJ Open Sport and Exercise Medicine*.

[41] Cusimano M. D., Nastis S., Zuccaro L. (2013). Effectiveness of Interventions to Reduce Aggression and Injuries Among Ice Hockey Players: A Systematic Review. *Canadian Medical Association Journal*.

[42] Tyler T. F., Nicholas S. J., Campbell R. J., Donellan S., McHugh M. P. (2002). The Effectiveness of a Preseason Exercise Program to Prevent Adductor Muscle Strains in Professional Ice Hockey Players. *The American Journal of Sports Medicine*.

[43] Edouard P., Dandrieux P. E., Blanco D. (2024). How Do Sports Injury Epidemiological Outcomes Vary Depending on Athletes’ Response Rates to a Weekly Online Questionnaire? An Analysis of 39-Week Follow-Up From 391 Athletics (Track and Field) Athletes. *Scandinavian Journal of Medicine and Science in Sports*.

[44] Reiman M. P., Agricola R., Kemp J. L. (2020). Consensus Recommendations on the Classification, Definition and Diagnostic Criteria of Hip-Related Pain in Young and Middle-Aged Active Adults From the International Hip-Related Pain Research Network, Zurich 2018. *British Journal of Sports Medicine*.

[45] Weir A., Brukner P., Delahunt E. (2015). Doha Agreement Meeting on Terminology and Definitions in Groin Pain in Athletes. *British Journal of Sports Medicine*.

